# A Novel Splicing Mutation in the *FBN2* Gene in a Family With Congenital Contractural Arachnodactyly

**DOI:** 10.3389/fgene.2020.00143

**Published:** 2020-02-28

**Authors:** Peiwen Xu, Ruirui Li, Sexin Huang, Menghan Sun, Jiaolong Liu, Yuping Niu, Yang Zou, Jie Li, Ming Gao, Xiaolei Li, Xuan Gao, Yuan Gao

**Affiliations:** ^1^ Center for Reproductive Medicine, Shandong University, Jinan, China; ^2^ National Research Center for Assisted Reproductive Technology and Reproductive Genetics, Jinan, China; ^3^ The Key Laboratory for Reproductive Endocrinology of Ministry of Education, Jinan, China; ^4^ School of Biological Science, University of California, Irvine, Irvine, CA, United States

**Keywords:** congenital contractural arachnodactyly, *FBN2* gene, novel splice mutation, in-frame deletion, whole exome sequencing

## Abstract

Congenital contractural arachnodactyly (CCA) is an extremely rare monogenic disorder in humans, and the prevalence of CCA is estimated to be less than 1 in 10,000 worldwide. CCA is characterized by arachnodactyly, camptodactyly, the contracture of major joints, scoliosis, pectus deformities, and crumpled ears. Mutations in *FBN2* (which encodes fibrillin-2) are responsible for causing this disease. A family with CCA was investigated in this study, and a novel variant, c.3724+3A > C (also identified as IVS28+3A > C), in *FBN2* was found in nine patients from the family but was not found in seven unaffected relatives. Reverse transcription-PCR (RT-PCR) and complementary DNA (cDNA) sequencing data showed that exon 28 was skipped in the *FBN2* gene. The *FBN2* c.3724+3A > C variant led to an in-frame deletion during transcription, which eventually triggered CCA in the Chinese family.

## Introduction

Congenital contractural arachnodactyly (CCA; MIM #121050), also known as Beals-Hecht syndrome, is an autosomal dominant connective tissue disorder (Lee et al., 1991; Putnam et al., 1995; Tuncbilek and Alanay, 2006) with extremely low morbidity compared with Marfan syndrome (MFS; MIM #154700) (Putnam et al., 1997), another hereditary connective tissue disease. CCA and MFS have many common clinical traits, including the so-called Marfanoid appearance, constituted by a tall, slender, asthenic appearance and skeletal features including arachnodactyly, dolichostenomelia, pectus deformities, and kyphoscoliosis (Jurko et al., 2013; Inbar-Feigenberg et al., 2014). However, most patients with CCA have “crumpled” ears, flexion contractures, and muscular hypoplasia (Meinecke, 1982; Maslen et al., 1997; Lavillaureix et al., 2017). These two similar syndromes are caused by mutations in two distinct genes both belonging to the fibrillin family, *FBN1* and *FBN2*, which encode fibrillin-1 and fibrillin-2, respectively (Frederic et al., 2009).

Up until August 10, 2019, only 116 different types of variants in the *FBN2* gene have been reported in the Human Gene Mutation Database (HGMD), and the clinical significance of some variations remains unknown. Pathogenic *FBN1* variants are spread throughout the gene, while the majority of *FBN2* mutations associated with CCA occur only in exons 24–35, which encode the calcium-binding epidermal growth factor-like (cbEGF) domains (Gupta et al., 2002; Zhou et al., 2018). These pathogenic variants reduce the amount of fibrillin-2 available to form microfibrils. Decreased microfibril formation reduces the elasticity of fibers, which leads to the symptoms of CCA (Davis and Summers, 2012; Sengle et al., 2015).

We found a novel variant in the *FBN2* gene, and it is likely the underlying etiology in the family with congenital contractural arachnodactyly presented here.

## Case Presentation

**Figure 1:**
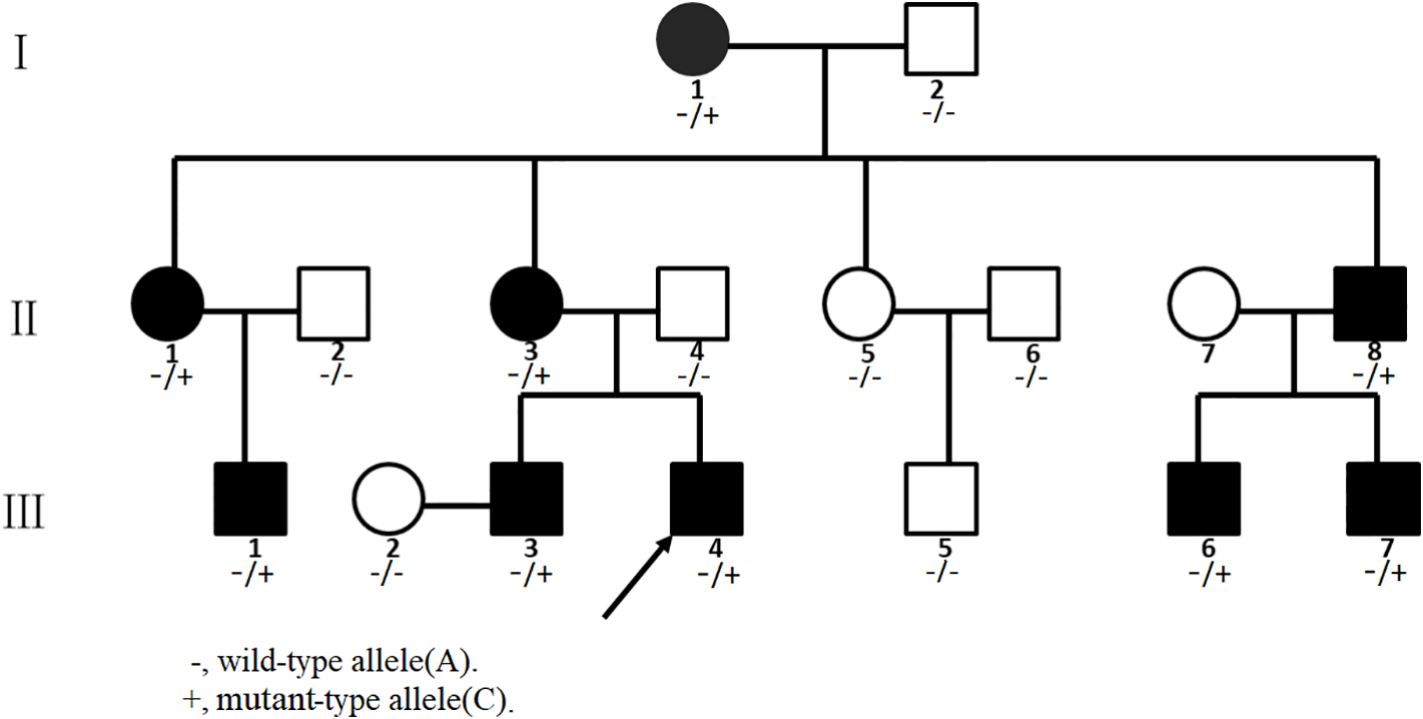
Pedigree of the Chinese congenital contractural arachnodactyly (CCA) family. Affected family members are denoted in black. The arrow indicates the proband.

We investigated a family with CCA with 16 individuals (**Figure 1**). The nine affected individuals were patients I-1, II-1, II-3, II-8, III-1, III-3, III-4, III-6, and III-7 at the time of the investigation (**Table 1**). Participants I-2, II-2, II-4, II-5, II-6, III-2, and III-5 were not diagnosed as having CCA. All participants had signed informed consent.

**Table 1:** Clinical features of the patients in pedigree.

Patient ID	Long slim limbs	Spider-like fingers	Thoracic malformation	Systemic musculoskeletal crouching	“Crumpled” ears	Stand on tiptoe
I-1	–	Y	–	Y	–	–
II-1	Y	Y	–	–	–	Y
II-3	–	–	Y	Y	–	–
II-8	Y	Y	–	–	–	–
III-1	Y	Y	Y	Y		Y
III-3	Y	Y	Y	Y	Y	Y
III-4	Y	Y	Y	Y	Y	–
III-6	Y	Y	–	–	–	–
III-7	Y	Y	Y	–	Y	–

Y, Yes, represents the appearance of the phenotype.

–, no obvious abnormal phenotype was observed.

**Figure 2:**
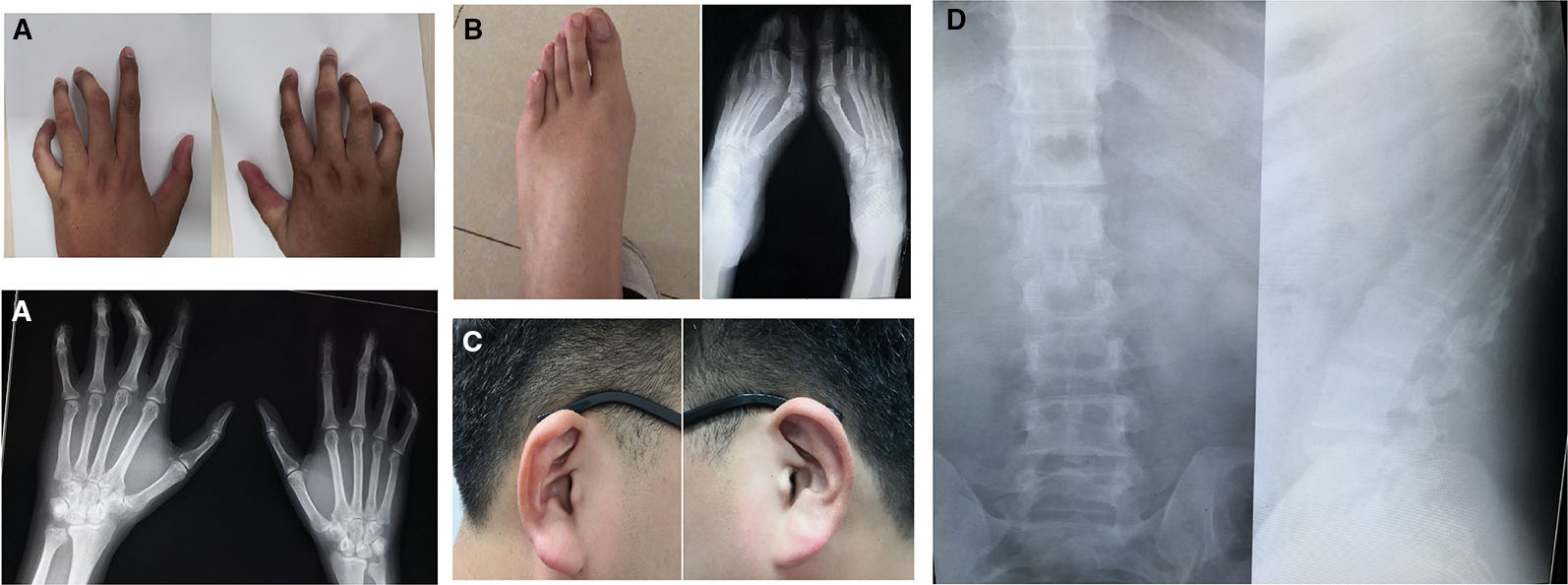
Clinical features of proband with congenital contractural arachnodactyly (CCA). **(A)** Photographs and x-ray plain film of fingers. **(B)** Photographs and x-ray plain film of toes. **(C)** Photographs of ears (after intervention). **(D)** Chest x-ray plain film.

The proband (III-4, **Figure 1**) is a Han Chinese male with a height of 190 cm and a weight of 110 kg, long slim limbs, long spider-like fingers, thoracic malformation, systemic musculoskeletal crouching, and “crumpled'' ears (**Figure 2**). He is unable to stand on tiptoe since his toes are abnormally long.

## Genetic Analysis

Five-milliliter blood samples were collected from every subject for genomic DNA extraction using the ZEESAN Blood Genomic DNA Mini Kit (ZEESAN, XiaMen, China). We performed whole-exome sequencing (WES) in the proband.

All variants were filtered through population databases including the 1000 Genomes Project (1KG), Exome Aggregation Consortium (ExAC), and gnomAD; only those variants with population frequencies less than 1/1,000 in all databases were counted. The pathogenicity was predicted by SIFT, PolyPhen 2, LRT, MetaSVM, MCAP, MutationTaster, as well as bioinformatics software that could predict splicing variant, including Human Splicing Finder (HSF), maximum entropy (MaxEnt), Berkeley Drosophila Genome Project (BDGP), and ESE finder. Possibly pathogenic variants were screened out *via* comparison with common disease databases (including ClinVar, HGMD, and HPO) and clinical phenotypes.

**Figure 3:**
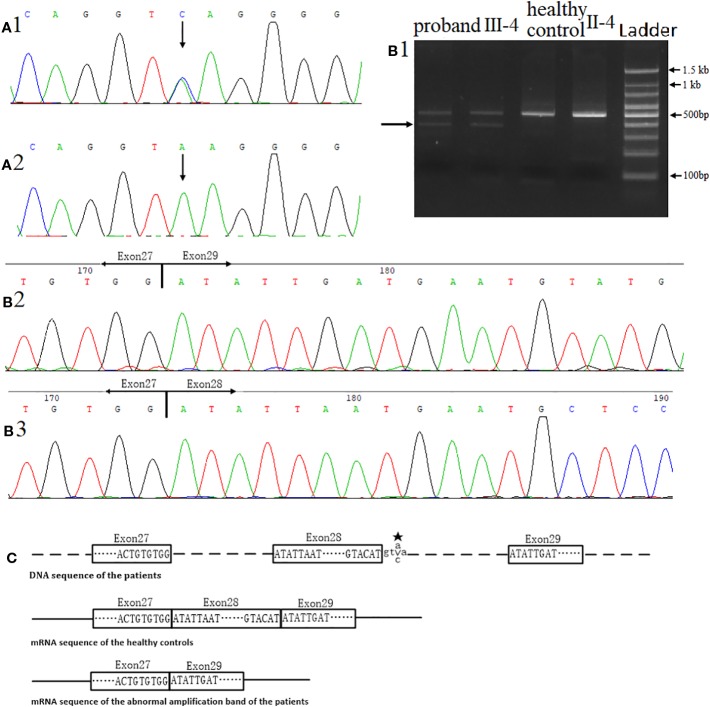
Identification of the novel variant in the *FBN2* gene. **(A)** Direct sequencing confirmed a heterozygous splice variant c.3724+3A > C in inton 28. **(A1)** DNA sequence of the proband. **(A2)** DNA sequence of the healthy control II-4. **(B)** Direct complementary DNA (cDNA) sequencing, **(B1)** Agarose gel electrophoresis for RT-PCR, the arrow indicated the unexpected band in the proband sample. **(B2)** cDNA sequence of the abnormal amplification band of the proband. **(B3)** cDNA sequence of the healthy control II-4. **(C)** The proband's in-frame deletion. ★-mutant base. Samples of proband and control were tested under the same conditions, including equal amounts of sample (peripheral blood and RNA) and experimental conditions.

We found a heterozygous noncanonical variant in intron 28 of the *FBN2* gene (hg19 chr5:127671677T/G, NM_001999.4, c.3724+3A > C, also identified as IVS28+3 A > C) in the proband (**Figure 3A1**). Sanger sequencing confirmed the variation. Genotyping of c.3724+3 was conducted using an automated ABI3730 DNA sequencer with the 5'-GGAGAACATTTTACTTCCTCGTG-3' (forward) and 5'-ACAATAACTGAGATCTGCCCCT-3' (reverse) primers. Sequencing results were analyzed by Chromas software (v 2.22) and NCBI BLAST tools. The variant was classified as likely pathogenic according to the ACMG guidelines (pathogenicity criteria PP1-S, PM2, PM4, PP4) (Richards et al., 2015). The variant has never been reported in the databases of Exome Sequencing Project (ESP), 1KG, ExAC, gnomAD, or database of SNP (dbSNP) (pathogenicity criterion PM2), and is predicted to affect the splicing by HSF. The reported variant was identified in heterozygous state in all nine affected individuals of the family, and was not observed in seven unaffected family members (pathogenicity criterion PP1-S) (**Figure 3A2**). Finally, the *FBN2* gene has been previously associated with CCA (pathogenicity criterion PP4).

We analyzed the effect of the splicing variant identified on the *FBN2* RNA product. Total RNA was extracted from the fresh blood samples using TRIzol (Takara Dalian, China). Complementary DNA (cDNA) synthesis was performed using the PrimeScript™ II 1st strand cDNA Synthesis Kit (Takara Dalian, China). PCRs were performed to amplify the *FBN2* cDNA (exon26-exon29, ~0.5 kb), the products of which were used for Sanger sequencing (BigDye^®^ Terminator Cycle Sequencing Kit, ABI3730, America). The primer sequences used in PCR were 5'-CAATACACCGGGCAGCTTTG -3' (forward) and 5'-GGCCGCCATCACAGATATCA -3' (reverse). The sequences were analyzed with Chromas software and NCBI BLAST.

When amplifying exons 26-29, we observed an additional PCR product in the proband cDNA sample, not present in the unaffected control cDNA sample (**Figure 3B1**). According to Sanger sequencing results, exon 28 of FBN2 (consisting of 126 nucleotides) was skipped in this shorter isoform. This leads to a predicted in-frame deletion of 42 amino acids in the FBN2 protein (pathogenicity criterion PM4) (**Figures 3B2, B3, C**).

## Discussion

We reported a novel splicing variant (c.3724+3A > C) in intron 28 of the *FBN2* gene that was discovered in a family with nine CCA patients.

**Figure 4:**
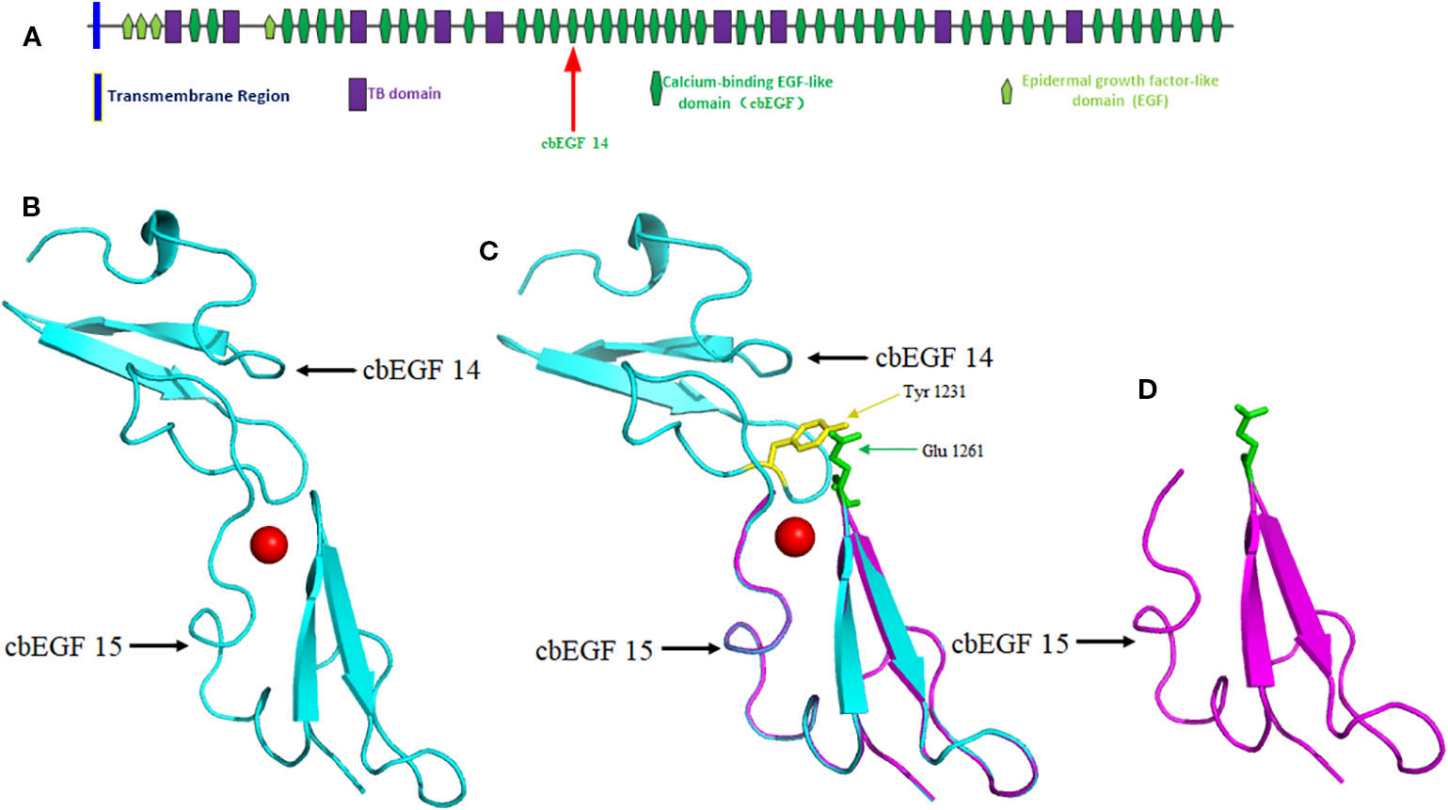
Domain structure of the *FBN2* protein and homology modeling of wild-type and mutant *FBN2* variants. **(A)** Domain 14 of cbEGF is located within putative structural domain of the fibrillin-2. **(B)** Modeled structure of cbEGF domains 14-15 of the fibrillin-2, the red ball means Ca^2+^. **(C)** Schematic of interdomain packing interactions for fibrillin-2 cbEGF 14-15, Tyr 1231 is highlighted in yellow and Glu 1261 is in green. The protein modeling is achieved by PyMOL Molecular Graphics System (Version 2.3.0) according to FBN1 structural coding (Downing et al., 1996; Smallridge et al., 2003). **(D)** Modeled structure of cbEGF domain 15 but without domain 14 of the fibrillin-2.

The native protein folding of each cbEGF-like domain is composed of six conserved cysteine residues, which form three disulfide bridges to maintain protein stability (Corson et al., 1993). Ca^2+^ binding in a negatively charged cavity improves folding stability and thus helps to secure the relative orientation of two neighboring cbEGF domains, consisting of a typical sheet-loop-sheet motif: two antiparallel beta-sheets bridged by a Ca^2+^ chelation loop (**Figure 4**) (Rao et al., 1995; Smallridge et al., 2003). In this report, we identified a splicing variant causing the skipping of exon 28 during messenger RNA (mRNA) synthesis. This is predicted to cause the in-frame deletion of 42 amino acids composing a cbEGF domain in fibrillin-2 protein, which diminishes the stability of an antiparallel beta-sheet and ultimately disrupts the folding of fibrillin-2 protein (**Figure 4**). As a result, the number of fibrillin-2 microfibers is cut down, so as to further reduce the amount of fibrillin-2 available to form microfibrils and finally lowered the elasticity of fibers, which triggers the congenital contracture. (Yuan et al., 1997; Isogai et al., 2003; Charbonneau et al., 2003; Lee et al., 2019).

MFS and CCA are derived from the dysfunction of the two proteins. MFS is more severe, especially in terms of the dilatation and interlining of the aorta as well as lethal cardiovascular lesions (Quondamatteo et al., 2002; Takeda et al., 2015). CCA, on the other hand, is distinct from MFS by crumpled ears, congenital contracture, and muscular maldevelopment (Meinecke, 1982; Yang et al., 1999). Nevertheless, their clinical features are largely similar. For instance, MFS and CCA patients both appear to be tall, emaciated and feeble and possess skeletal features including extremely long fingers, toes, and limbs, pectoralis malformations, and kyphosis, namely, the Marfan appearance (Mariencheck et al., 1995). It is difficult to differentiate MFA from CCA only on the basis of clinical symptoms (Gupta et al., 2004); thus, genetic analysis is a more reliable diagnostic tool.

*FBN2* mutations are supposed to alter fibrillin-2 central domain structures by changing key binding sites (which are cysteines), deleting the whole domains, or by inserting new glycosylation sites into the domains. Most mutations discovered in *FBN2* are located in the repeating sequence region. You *et al*. reported a heterozygous missense variant in the cbEGF functional domain, which generated folding instability in the fibrillin-2 cbEGF domain and thus triggered CCA symptoms (You et al., 2017). Putnam *et al*. reported a branch point sequence variant that led to a splicing error in exon 29 of the *FBN2* gene and consequently induced CCA (Putnam et al., 1997), this variant is similar to that found in the study reported here, which resulted in the loss of an exon, leading to the loss of a cbEGF domain. Conversely, a missense mutation that alters consensus sequence amino acids in cbEGF domains is the major potential molecular defect in MFS patients caused by *FBN1* mutations.

To conclude, the presented study reports a novel FBN2 variant, c.3724+3A > C (also as IVS28+3 A > C), which was identified in a Chinese family with CCA using NGS and Sanger sequencing. All of the factors together, including the patient phenotypes, functional alterations, and *in-silico* prediction analyses, indicated that the variant was a pathogenic variant that resulted in CCA. This variant enriches the spectrum of *FBN2* mutations and should be considered as a genetic cause for CCA for future genetic counseling and prenatal diagnosis.

## Data Availability Statement

The datasets generated for this study can be found in the NCBI PopSet, MN510778, MN510779.

## Ethics Statement

The studies involving human participants were reviewed and approved by The Ethics Committee of the Reproductive Medical Hospital Affiliated to Shandong University. The patients/participants provided their written informed consent to participate in this study. Written informed consent was obtained from the individual(s) for the publication of any potentially identifiable images or data included in this article.

## Author Contributions

PX was involved in the study design, data analysis, and manuscript drafting. RL, SH, JLiu, and YN did the clinical assessment and recruitment of the patients and their family members. YZ, JLi, MG, and XL performed the experiments and critically reviewed the manuscript. MS were involved in the study design and critical evaluation of the manuscript. XG and YG conceived the study and wrote the manuscript.

## Funding

The study was funded from the National Key Research and Development Program of China (2018YFC1003100; 2018YFC1004900); Natural Science Foundation of Shandong Province (ZR2018PH006; ZR2018MC014), Key Research and Development Program of Shandong Province (2017G006035).

## Conflict of Interest

The authors declare that the research was conducted in the absence of any commercial or financial relationships that could be construed as a potential conflict of interest.
